# Supplementary material for: Hands Up Program: Results of a feasibility study of a randomized controlled trial of a bone health exercise and education program for adults aged 50–65 post distal radius fracture
Source: PLoS One. 2024 Nov 5;19(11):e0313013. doi: 10.1371/journal.pone.0313013 (PMC11537389; doi:10.1371/journal.pone.0313013)
Supplement: S3 File — (PDF) [file pone.0313013.s004.pdf]

BMJ Open

Hands-Up program: protocol for a feasibility randomised controlled trial of a combined 6-week exercise and education intervention in adults aged 50–65 with a distal radius fracture

Christina Ziebart [1] Joy MacDermid,[1,2] Dianne Bryant,[3] Mike Szekeres,[2] Nina Suh[2]

To cite: Ziebart C, MacDermid J, Bryant D, *et al*. Hands-Up program: protocol for a feasibility randomised controlled trial of a combined 6-week exercise and education intervention in adults aged 50–65 with a distal radius fracture. *BMJ Open* 2021;**11**:e046122. doi:10.1136/bmjopen-2020-046122

► Prepublication history and additional supplemental material for this paper are available online. To view these files, please visit the journal online (http://dx.doi.org/10.1136/bmjopen-2020-046122).

Received 21 October 2020
Accepted 15 July 2021

Check for updates

© Author(s) (or their employer(s)) 2021. Re-use permitted under CC BY-NC. No commercial re-use. See rights and permissions. Published by BMJ.

[1]Department of Physical Therapy, Western University, London, Ontario, Canada
[2]St Joseph's Health Centre, London, Ontario, Canada
[3]Faculty of Health Sciences and Schulich School of Medicine and Dentistry, University of Western Ontario, London, Ontario, Canada

Correspondence to
Dr Christina Ziebart; cziebart@uwo.ca

ABSTRACT

Introduction This pilot study will determine the feasibility of recruitment, retention and adherence for the online combined exercise and education programme (Hands-Up) compared with usual care.

Methods and analysis The proposed randomised controlled trial will be a pilot feasibility study with a 1:1 randomisation to the Hands-Up programme or usual physical therapy. The programme setting is an online home exercise and education program. Outcome assessments will take place at the Roth|McFarlane Hand and Upper Limb Clinic in London, Ontario, which is a tertiary care academic centre. 74 people aged 50–65 years after distal radius fracture will be recruited. Purposeful recruitment will be conducted to include a sufficient number of men in the study. The Hands-Up program includes both exercise and education. Participants will engage in approximately 45 min of a full-body home exercise routine followed by 30 min of education in an online program. The educational modules will focus on fall prevention, nutrition for osteoporosis and learning about osteoporosis. Participants will engage in the program online, twice a week for 6 weeks. Outcomes will be assessed at baseline, 6 weeks and 3, 6 and 12 months. The primary outcome of the study is feasibility. The study will be considered feasible if participants attend >60% of the sessions, 74 participants are enrolled in 12 months and >75% of participants attend the final study visit. For the secondary outcome, physical and self-report outcomes will be assessed.

Ethics and discussion This study has been approved by the institutional ethics review board. The viability of an online exercise and education program for people after distal radius fracture between the ages of 50 and 65 will be evaluated in an attempt to help better prevent, diagnose and manage osteoporosis. This study will be disseminated through peer-reviewed publications, international conferences and social media.

Trial registration number NCT03997682.

Strengths and limitations of this study

- ► This study is strengthened by being offered online, allowing for better accessibility for the patients, particularly in a time when in-person sessions are compromised with COVID-19.
- ► This study is aiming for a relatively large sample size for a pilot study, which will provide insight into the feasibility of this program long term.
- ► This study is a pilot study, limiting the ability to determine exercise effects related to strength and endurance.
- ► This study is strengthened by a strong knowledge translation plan to disseminate the results.
- ► Limitations include decreased external validity to only English-speaking countries and challenges in assessing medium-term and long-term effects of the trial.

INTRODUCTION

Distal radius fractures (DRF) have the highest incidence of any fractures[1] and account for one-sixth of all emergency department visits.[2 3] Consequences related to DRF are pain and functional limitations, which have been reported to occur in the acute and chronic phases of recovery,[4–6] with 16% of people with a DRF reporting pain up to 1 year after fracture.[7 8] There have been several factors reported to be associated with negative outcomes after a DRF, such as higher age,[9] female sex[9] and joint involvement during play and work (i.e. jobs requiring high demands from the hand and wrist typically have worse outcomes than jobs that require minimal use of the hand and wrist)[4 7] are potentially related to sustained feelings of pain and disablement. Several studies have looked at the effect of DRF pain across a variety of age categories and found statistically significant differences in those aged above 65 and below 65.[9] In an observational cohort study of over 1500 participants the highest incidence of fracture occurred in the 51–65 age

group, with men reporting the highest pain in that age group.[10] It was hypothesised that the high incidence of fractures found in those people aged 51–65 is likely due to changes in bone mineral density, potentially caused by osteoporosis.

Osteoporosis is a bone disease that occurs from a decrease in bone mineral density, increasing the risk of non-traumatic fractures. Fractures in the wrist, hip and vertebrae are the most common. However, a fracture at the wrist is usually the first indication of the presence of osteoporosis.[11] People with osteoporosis who are not taught to modify their lifestyle, specifically through exercise, nutrition and fall prevention strategies, are more likely to suffer more debilitating fractures of the hip and spine later in life.[12] Current wrist fracture rehabilitation focuses on restoration of joint mobility and hand function,[11] and focuses on those over the age of 65. However, those 50–65 years of age represent the largest group of people presenting with wrist fractures.[10] They may require more targeted rehabilitation approaches since their bone health and activity levels are different from older adults. Further, early intervention provides the opportunity for preventing future debilitating fractures of the spine or hip.

There is little information conveniently accessible to people related to exercise, nutrition and fall prevention to reduce the risk of osteoporotic fractures.[13] The goal of this research is to develop and test a novel exercise and education group intervention (Hands-Up) that will incorporate education on bone health, prevention guidelines, nutrition and fall prevention, with an online exercise class and a structured virtual home safety assessment. Specifically, is the Hands-Up program a feasible intervention for patients aged 50–65 with a low-impact DRF? Secondarily, will the Hands-Up programme potentially produce better, range of motion (ROM), strength and reduce pain than usual physical therapy for patients aged 50–65 with a low-impact DRF when tested in a fully powered randomised clinical trial? This multimodal intervention will target the major risk factors for secondary fractures, to enhance functional recovery and health following a DRF, compared with usual physical therapy for wrist fractures.

METHODS AND ANALYSIS

Study setting

This study is organised by the Roth|McFarlane Hand and Upper Limb Clinic (RM-HULC) in London, Ontario. RM-HULC is a tertiary care centre specialising in patients with complex conditions affecting the hand, wrists, elbows and shoulders. RM-HULC serves over 40 000 patients per year, making it a resource for recruiting patients with a DRF. The program itself will take place online. Participants will engage in this program from their home, online.

Trial design

This study will be a single-blinded, non-inferiority randomised controlled trial comparing an online twice-weekly exercise and education session to usual physical therapy treatment for people with a DRF. There will be a 1:1 allocation ratio. A web-based randomisation software will be used to generate allocation assignments stratified by gender. Allocation will be concealed from therapists and patients until the first visit, and a blinded evaluator will perform all patient outcome assessments (CZ). This is protocol version 2 from September 2020.

Participants

We will recruit volunteers aged 50–65 years, who are 6–10 weeks after wrist fracture, from the RM-HULC in London, Ontario. Participants will not be required to have preintervention osteoporosis or osteopenia, but we will be aiming to enrol participants with a low-impact DRF, to target participants at high risk of developing osteoporosis in the future. Participants will have had a recent DRF and will be cleared from exercise by their treating physician. We are aiming to enrol a total of 74 participants, 32 participants in the Hands-Up intervention group and 32 participants in the control group. The sample size was designed based on multiple papers and criteria for pilot studies[14 15] and to provide sufficient data to pilot our disaggregated sex/gender-based analyses (SGBA) plan.[16] Given the predominance of women in this population, we will specifically recruit men to ensure that at least 40% of participants are men, to support SGBA.

The inclusion criteria will be:

- Patients attending the RM-HULC.
- Patients who have had a DRF within the last 6–10 weeks.
- Aged 50–65.
- Able to speak and understand English.
- Able to provide informed consent.

Exclusion criteria will be:

- Any contraindications to exercise.
- Progressive neurological disorders that would affect study participation.
- Unable to stand or walk independently.
- Unable to provide consent.

Patient and public involvement

Previous work by our laboratory group surveyed people with osteoporosis to better understand their exercise preferences. The feedback from that survey helped to design the research question and design of the study.[17] People with osteoporosis were consulted on the education materials and the exercise programme to determine whether the education and exercise were relevant and thorough. Participants will be asked to discuss the patient burden of the study during their study participation. A substudy will be conducted to assess the usability and participant engagement with the intervention.

Intervention

The participants will be asked to participate in the online sessions twice weekly for 6 weeks, which will consist of 45

Ziebart C, et al. BMJ Open 2021;11:e046122. doi:10.1136/bmjopen-2020-046122

Table 1 Example exercise programme for participants to use to track their exercise and follow through the circuit

Date: SAMPLE

Begin with a 5 min warm-up, and always start with lighter weights and you can always go higher. Aim for a weight that you can do 8–12 repetitions. The 12th repetition should be challenging without breaking form. If you can get to 15 repetitions without struggle, then increase the weight.

Exercise	Set	Reps	Weight	Set	Reps	Weight	Notes
Squat							
Hip hinge							
Reverse lunge							
Single leg deadlift							
Inclined push-ups							
Single arm supported row							
Upright row							
Lat pulldown							
Band pull apart							
Band external rotation							
Tandem stand							
Single leg stand							
Take some time to do your hand therapy exercises and some stretches.							

min of a prerecorded, guided online exercise program and 30 min of prerecorded education modules. The online exercise program will consist of three components:

- Upper extremity mobility and strength exercises.
- Lower extremity mobility and strength exercises.
- Static and dynamic balance training (table 1).

Exercise program

Participants will be guided through a 45 min exercise program. In the first session, the participants will be asked to go through tutorial videos teaching them how to perform each exercise. The subsequent sessions will be a guided 45 min class of a prerecorded exercise program, where the participants can follow along with the video. The participants will track their progress on a program sheet, recording the number of sets and the number of repetitions completed for that session, and any modifications made to the exercises. An example exercise program is presented in table 1. The exercises will be progressed after 3 weeks, where the participants will be able to follow along with a second 45 min video demonstrating these progressed exercises. An online forum will be set up and monitored by a physical therapist to answer any questions that arise during the exercise program.

Education sessions

Immediately after the exercise session, participants will be encouraged to participate in a 30 min educational session. The educational sessions will be prerecorded videos. The topics will cover bone health principles, nutrition for bone health, osteoporosis practice guidelines, ways to self-monitor balance and lower extremity strength, impacts of physical activity, home hazard detection, hazards at work and in the community, postural effects on bone loading and fracture risk and integrating physical activity in daily life. Nutritional education will emphasise the importance of calcium and vitamin D, sources of both dairy and dairy-free calcium, vitamin D, the importance of protein, and meat and meat-free sources of protein. Although the participants will be encouraged to listen to the videos immediately after they exercise, they have flexibility when they listen to the modules. They will be encouraged to listen to them in order and over the 6-week intervention timeline. Participants will be asked to post any question on the online forum, which will be monitored, and the questions will be answered by a physical therapist.

The fall hazard detection will use a fall hazard checklist to identify home and workplace fall hazards and assist participants in identifying fall hazards in their home environments. Patients will complete a checklist of common home hazards and then record themselves navigating their home and talk aloud to identify potential hazards. The videos will be submitted online and reviewed by a physical therapist. The last session of the program will allow the participant to discuss the program with the physical therapist. Patients randomised to regular care and the Hands-Up program will both receive therapy as directed by their treating therapist and complete all study outcome measures. Participants in the standard treatment group will receive access to the online materials after their study participation.

Setting and supervision

All exercise sessions will take place at home. The participants will be asked to complete an exercise tutorial prior to beginning the guided online program. There will be safety precaution statements and an online forum to allow for the participants to ask questions. The videos are not live, but a physical therapist will be frequently monitoring

the forum to answer any questions. Adverse events will be noted at the outcome assessment visits and monitored on the online forum. The student investigator will be conducting the outcome assessments and will conduct the assessments on all the participants, which will also take place at RM-HULC.

Website

The participants will engage in the online program through a website hosted by Wix.com, accessed with the URL: handsup-program.com. To reduce the risk of contamination of the study, a login and password are required to enter the website. Each participant will create their own login information. Security and privacy information can be accessed through the Wix website: https://supportwixcom/en/article/about-domain-privacy. In the creation of the Wix website, we have opted to enhance the domain security to reduce the risk of identity theft. However, to make sure that all the participants can access the website it is publicly accessible. A user login helps to reduce the risk of the public accessing online forum questions; however, other participants in the study will be able to view the forum. Participating in the forum is voluntary, and if participants do not feel comfortable, they do not have to participate. Instead, participants canprivately email the lead student investigator directly.

Goals and exercise modes

The program was designed with four main goals in mind: (1) to improve upper body strength and mobility; (2) to improve lower body strength and mobility; (3) to improve balance; and (4) to improve functional outcomes of the wrist due to the DRF. The current study is assessing feasibility, and if the study is feasible a larger trial will be designed to determine whether these goals are met.

Frequency and duration

Participants will be asked to participate in the exercise and education class twice weekly for 6 weeks. Each session will last 75 min, with 45 min allocated to exercise and 30 min to education. Participants will not be explicitly encouraged to exercise beyond the program; however, they will be provided with a diary to track any exercise they might be doing outside of the program.

Intensity

For the upper and lower body strengthening the participants will use body weight, or a resistance band. It will be up to the participants' discretion to determine their appropriate intensity. Since the participants will be provided resistance bands but no weights, we will ask them to increase intensity by increasing the number of reps, or sets, or the resistance on the band. It will be emphasised that the participants prioritise good form rather than high repetitions or increasing resistance. The online exercise program will be set up to have the participants complete three sets of 8–10 repetitions of exercises targeting the major muscle groups. The exercises can be progressed by increasing the resistance or by increasing the number of repetitions. Balance exercises will be held for 20–60 s, progressively increasing the difficulty by reducing the base of support, adding a mental challenge or affecting the sensory system (eg, closing their eyes). Participants will be coached on how to self-progress.

Time

The intervention group will be asked to participate in the 6-week program, but after that participation they will not be asked to continue the exercise program. However, we will be tracking their activity through a log for 1 year.

Comparator

The control group will not receive the exercise and education portion of the intervention. They will be asked to attend a baseline visit for measurement of outcomes, and report back at 6 weeks, month 3, month 6 and month 12 to repeat the outcome assessments. Participants in the control group will be attending the usual physical therapy care that they would receive after a DRF. The control group will receive access to the online materials at the end of the 12-month study participation.

Data collection and management

A study manual describing the operating procedure, consent forms, scripts, data forms and checklists are in the Hands-Up Study Manual. The lead student investigator (CZ) will manage the trial, be the point of contact for the participants and allocate them to their group. A research assistant will obtain consent and perform the outcome assessments. The lead student investigator will not be blinded to study allocation, but the research assistant collecting the data will be blinded. Participants will be able to opt out of assessments and will be asked to complete the questionnaires electronically through a secure database. A secure online data management system will be used and monitored by the lead student investigator. A copy of the letter of information and consent is provided in online supplemental file 1.

Primary outcome: feasibility, recruitment and retention

For the primary outcome, the intervention will be considered feasible if participants complete >60% of the prescribed classes (two classes per week for 6 weeks).[18] Recruitment will be determined as feasible if we are able to recruit 74 patients within 12 months. Retention rates will be considered feasible if greater than 75% of participants attend the final study visit[19] (figure 1).

Secondary outcomes

For the secondary outcome, functional assessment measures will include:

- Physical performance: grip strength, Step Test, Biodex Balance Test and Five Times Sit to Stand Test.
- Self-report: physical activity (rapid assessment of physical activity. personalized exercise questionnaire), self-reported bone health behaviours, hand function (ROM, Patient-Rated Wrist Evaluation Questionnaire).

	STUDY PERIOD						
	Enrolment	Allocation	Post-allocation				Close-out
TIMEPOINT	*Baseline-t_1*	0	*t_1= 6 weeks*	*t_2= 3 months*	*t_3= 6 months*	*t_4= 12 months*	*t_x*
ENROLMENT:							
Eligibility screen	X						
Informed consent	X						
Allocation		X					
INTERVENTIONS:							
[Hands- Up Program]			x	←	—	→	
[Control]			←	—	—	→	
ASSESSMENTS:							
[Feasibility, retention, recruitment]	X	X	X			X	X
[Physical performance outcomes]	X		X	X	X	X	
[Self-Report outcomes]	X		X	X	X	X	
[Bone Density Scan]	X				X	X	

Figure 1 The schedule of enrolment, interventions and assessments for the Hands-Up pilot randomised controlled trial.

- ► Bone density measure: dual energy X-ray absorptiometry (DXA), measured at the wrist, femur and total body.

Knowledge will be assessed with knowledge tests on bone health behaviours.

These assessments will be measured at baseline, 6 weeks and 3, 6 and 12 months following wrist fracture, except for the DXA, which will be measured at baseline, 6 months and 12 months. The participants will complete the standardised self-reporting tools that will assess adherence to the programme (figure 1).

Recruitment

Participants will be recruited from the RM-HULC. Surgeons will be used to help notify the lead student investigator for an eligible participant after approaching the participant, explaining the study, asking if they are willing to participate and providing the participant with the letter of information and consent. The lead student investigator will then approach the participant to set up the initial visit. It is the goal to have 74 participants enrolled in the study within 1 year of starting recruitment.

Strategies to enhance retention

Participants will be asked to return for assessment visits at baseline, 6 weeks and 3, 6 and 12 months. Participants will be called 1 month prior to their scheduled assessment time to set up the visit and an additional reminder phone call will be made the day prior to the visit.

Sample size estimation

A total of 74 participants, 32 in the Hands-Up group and 32 in the control group, is the sample size goal. The sample size was designed based on multiple papers and criteria for pilot studies,[14 15] and to provide sufficient data to pilot our disaggregated SGBA plan.[16] Given the predominance of women in this population we will specifically recruit men to aim for 40% of men participants, t supporting SGBA recommendations.

Analysis

The quantitative variables will be presented as means and SD, and the qualitative variables as numbers and percentages. Feasibility outcomes will be presented as number of participants meeting the a priori definitions. To compare the baseline data of gender and dominant hand of the participant, χ^2 tests will be used. A preliminary analysis (general linear modelling) of between-group differences, disaggregated by gender including associated CIs to determine the range of potential treatment effects (table 2).

ETHICS AND DISSEMINATION

We are proposing a feasibility study improving outcomes for adults after DRF. This intervention will combine exercise and osteoporosis education. This study was designed as a pilot study, a smaller scale study, to determine whether this intervention is feasible and practical for a future larger scale study. The study was designed in such a way that the intervention will be closer on the scale of pragmatic rather than explanatory as a hope of the intervention being adapted into practice. A larger trial could be expanded into other cities, provinces or countries, and in a variety of treatment centres.

This will be the first study to address adults aged 50–65 with a DRF by providing them with a whole-body exercise program and osteoporosis-related education, and the first programme to offer this type of programme online. This study chooses to cater the education towards osteoporosis because osteoporosis is a very prevalent disease[20] with a large gap in care in this age group.[13] Specifically, a DRF is often the first sign of someone having osteoporosis[21–23]; however, it often takes more than one fracture before osteoporosis screening or treatment begins. Meanwhile, the 50–65 years old age category is at high risk of developing osteoporosis.[20 24 25] If we are able to teach them non-pharmacological management, they may be able to be more autonomous in their care and potentially prevent future more debilitating fractures such as of the hip or spine.

This study was designed to be presented in English, potentially limiting the external validity of the study. However, over 200 million people suffer from osteoporosis worldwide,[26] indicating a need for preventative management strategies. Future iterations of this programme could include translations to target a larger proportion of those individuals affected by osteoporosis. Unfortunately, it will be challenging to determine the medium-term and long-term effects of the programme, as the goal is to prevent fractures.

Ethics and confidentiality

The research will be conducted according to the Tri-Council Policy Statement and in accordance with the University of Western Ontario and Lawson Health

Table 2 Variable, hypotheses, outcomes and methods of analysis

Variable/outcome	Hypothesis	Outcome measure	Method of analysis
**Primary**			
Recruitment	We will recruit 74 participants.	Number of participants recruited	Descriptive statistics
Retention	We will retain 75% of our sample.	Number of participants completing the final study visit	Descriptive statistics
Adherence	The average proportion of exercise sessions completed will be equal or greater than 60%.	The average proportion of completed exercise sessions	Descriptive statistics
**Secondary**			
Physical performance	The intervention will have improved outcomes relative to control.	► Grip strength<br>► Step Test<br>► Biodex Balance Test<br>► Five Times Sit to Stand Test	► t-test comparing baseline to final study visit<br>► t-test comparing final visit of intervention and control<br>► Logistical regression measuring change over time
Falls	The intervention group will have fewer falls.	Number of self-reported falls<br>Number of fallers	► Number of fallers: relative risk<br>► Fall rate: binomial regression
Fear of falling	The intervention group will have a reduced fear of falling.	Questionnaire	► t-test comparing baseline to final visit and intervention to control<br>► Regression to see change over time
Physical activity (RAPA)	The intervention group will have higher reported activity levels.	Questionnaire	► t-test comparing baseline to final visit and intervention to control<br>► Regression to see change over time
Self-reported bone health behaviours (PEQ)	The intervention group will have improved bone health behaviours.	Questionnaire	► t-test comparing baseline to final visit and intervention to control<br>► Regression to see change over time
Range of motion	The intervention group will have better range of motion.	Wrist goniometer	► t-test comparing baseline to final visit and intervention to control<br>► Regression to see change over time
Patient-rated wrist evaluation	The intervention group will have reduced pain and improved function.		► t-test comparing baseline to final visit and intervention to control<br>► Regression to see change over time
Bone density	The bone density will stay the same in the intervention group.	DXA	
Osteoporosis knowledge	The intervention group will have improved osteoporosis knowledge.	Knowledge test	► t-test comparing baseline to final visit and intervention to control<br>► Regression to see change over time

DXA, dual energy X-ray absorptiometry; PEQ, Personalized Exercise Questionnaire; RAPA, rapid assessment of physical activity.

Research. The study has received approval from the Research Ethics Board at the University of Western Ontario. A trial run of the study was conducted in August 2020 with a participant volunteer. Amendments will be submitted to the ethics committee by the lead student investigator. Participants will be assigned an ID to be used on the forms and in the data management system. Deidentified data will be stored in a secure area of RM-HULC. Hard copies of records with personal identifiers will be kept separately from the data. Data will be entered into the data management system by the lead student investigator.

Dissemination

Publishing the results will be a key strategy to provide knowledge users with the most up-to-date research for wrist fracture management in middle-aged adults. The program will be provided freely through open access to ensure academics and clinicians are informed of the study results. The results will also be disseminated to

academic knowledge users through national and international conference presentations. Workshops will be held to teach clinicians the results of the study, to inform their current practice. The lead student investigator will reach out to an osteoporosis exercise training program, BoneFit, to inform physical therapy practice on managing adults in the early stages of osteoporosis.

Results related to the exercise program, key nutritional points and fall prevention strategies will be made public through YouTube, also allowing for an indication of prograe uptake. Public access through YouTube will provide knowledge from the study results to individuals with osteoporosis and allow them to begin modifying their lifestyle. We will promote the study through social media to be able to reach a wider breadth of interested people.

We will also be targeting patient support groups such as the Canadian Osteoporosis Patient Network and translating the results through Osteoporosis Canada. Finally, a website related to this study will be created after the programe is complete to allow the patients and clinicians to have continued access to the resources.

Acknowledgements CZ is supported by the CIHR Doctoral Award. JM was supported by a CIHR Chair in Gender, Work and Health and the Dr James Roth Research Chair in Musculoskeletal Measurement and Knowledge Translation.

Contributors All authors have read and approved the manuscript. CZ prepared the manuscript, which was edited by the remaining authors (JM, DB, MS, NS). The protocol was developed with input from all contributing authors (CZ, JM, DB, MS, NS).

Funding The authors have not declared a specific grant for this research from any funding agency in the public, commercial or not-for-profit sectors.

Competing interests None declared.

Patient consent for publication Not required.

Provenance and peer review Not commissioned; externally peer reviewed.

Supplemental material This content has been supplied by the author(s). It has not been vetted by BMJ Publishing Group Limited (BMJ) and may not have been peer-reviewed. Any opinions or recommendations discussed are solely those of the author(s) and are not endorsed by BMJ. BMJ disclaims all liability and responsibility arising from any reliance placed on the content. Where the content includes any translated material, BMJ does not warrant the accuracy and reliability of the translations (including but not limited to local regulations, clinical guidelines, terminology, drug names and drug dosages), and is not responsible for any error and/or omissions arising from translation and adaptation or otherwise.

Open access This is an open access article distributed in accordance with the Creative Commons Attribution Non Commercial (CC BY-NC 4.0) license, which permits others to distribute, remix, adapt, build upon this work non-commercially, and license their derivative works on different terms, provided the original work is properly cited, appropriate credit is given, any changes made indicated, and the use is non-commercial. See: http://creativecommons.org/licenses/by-nc/4.0/.

ORCID iD

Christina Ziebart http://orcid.org/0000-0002-3667-4133

REFERENCES

1 MacIntyre NJ, Dewan N. Epidemiology of distal radius fractures and factors predicting risk and prognosis. *J Hand Ther* 2016;29:136–45.
2 Bonafede M, Espindle D, Bower AG. The direct and indirect costs of long bone fractures in a working age US population. *J Med Econ* 2013;16:169–78.
3 Kilgore ML, Morrisey MA, Becker DJ, *et al*. Health care expenditures associated with skeletal fractures among Medicare beneficiaries, 1999-2005. *J Bone Miner Res* 2009;24:2050–5.
4 MacDermid JC, Roth JH, McMurtry R. Predictors of time lost from work following a distal radius fracture. *J Occup Rehabil* 2007;17:47–62.
5 Brenneman SK, Barrett-Connor E, Sajjan S, *et al*. Impact of recent fracture on health-related quality of life in postmenopausal women. *J Bone Miner Res* 2006;21:809–16.
6 Hallberg I, Rosenqvist AM, Kartous L, *et al*. Health-related quality of life after osteoporotic fractures. *Osteoporos Int* 2004;15:834–41.
7 MacDermid JC, Roth JH, Richards RS. Pain and disability reported in the year following a distal radius fracture: a cohort study. *BMC Musculoskelet Disord* 2003;4:1–13.
8 Moore CM, Leonardi-Bee J. The prevalence of pain and disability one year post fracture of the distal radius in a UK population: a cross sectional survey. *BMC Musculoskelet Disord* 2008;9:1–10.
9 Mehta SP, MacDermid JC, Richardson J, *et al*. Baseline pain intensity is a predictor of chronic pain in individuals with distal radius fracture. *J Orthop Sports Phys Ther* 2015;45:119–27.
10 Ziebart C, MacDermid JC, Suh N. Effects of gender, age, and time on wrist pain up to two years following distal radius fracture. *Crit Rev Phys Rehabil Med* 2020;32:85–96.
11 Dewan N, MacDermid JC, Grewal R, *et al*. Recovery patterns over 4 years after distal radius fracture: descriptive changes in fracture-specific pain/disability, fall risk factors, bone mineral density, and general health status. *J Hand Ther* 2018;31:451–64.
12 Kanis JA. Diagnosis of osteoporosis and assessment of fracture risk. *Lancet* 2002;359:1929–36.
13 Ziebart C, McArthur C, Lee L, *et al*. "Left to my own devices, I don't know": using theory and patient-reported barriers to move from physical activity recommendations to practice. *Osteoporos Int* 2018;29:1081–91.
14 Hertzog MA. Considerations in determining sample size for pilot studies. *Res Nurs Health* 2008;31:180–91.
15 Leon AC, Davis LL, Kraemer HC. The role and interpretation of pilot studies in clinical research. *J Psychiatr Res* 2011;45:626–9.
16 Johnson JL, Greaves L, Repta R. Better science with sex and gender: facilitating the use of a sex and gender-based analysis in health research. *Int J Equity Health* 2009;8:14–11.
17 Ziebart CMJ, Bryant D, Szekeres M, *et al*. Exercise preferences for people with osteoporosis, identifying barriers, facilitators, needs and goals of exercise. *J Osteop Physic Act* 2020;8:1–7.
18 Gold DT, Shipp KM, Pieper CF, *et al*. Group treatment improves trunk strength and psychological status in older women with vertebral fractures: results of a randomized, clinical trial. *J Am Geriatr Soc* 2004;52:1471–8.
19 Papaioannou A, Adachi JD, Winegard K, *et al*. Efficacy of home-based exercise for improving quality of life among elderly women with symptomatic osteoporosis-related vertebral fractures. *Osteoporos Int* 2003;14:677–82.
20 Leslie WD, Morin SN. Osteoporosis epidemiology 2013: implications for diagnosis, risk assessment, and treatment. *Curr Opin Rheumatol* 2014;26:440–6.
21 Vogt MT, Cauley JA, Tomaino MM, *et al*. Distal radius fractures in older women: a 10-year follow-up study of descriptive characteristics and risk factors. The study of osteoporotic fractures. *J Am Geriatr Soc* 2002;50:97–103.
22 Mallmin H, Ljunghall S. Distal radius fracture is an early sign of general osteoporosis: bone mass measurements in a population-based study. *Osteoporos Int* 1994;4:357–61.
23 Ring D, Jupiter JB. Treatment of osteoporotic distal radius fractures. *Osteoporos Int* 2005;16:S80–4.
24 Papaioannou A, Morin S, Cheung AM, *et al*. 2010 clinical practice guidelines for the diagnosis and management of osteoporosis in Canada: summary. *CMAJ* 2010;182:1864–73.
25 Prior JC, Langsetmo L, Lentle BC, *et al*. Ten-year incident osteoporosis-related fractures in the population-based Canadian multicentre osteoporosis study - comparing site and age-specific risks in women and men. *Bone* 2015;71:237–43.
26 Vijayakumar R, Büsselberg D. Osteoporosis: an under-recognized public health problem: local and global risk factors and its regional and worldwide prevalence. *J Local Global Health Sci* 2016;2016:2.
