# Supplementary material for: Hands Up Program: Results of a feasibility study of a randomized controlled trial of a bone health exercise and education program for adults aged 50–65 post distal radius fracture
Source: PLoS One. 2024 Nov 5;19(11):e0313013. doi: 10.1371/journal.pone.0313013 (PMC11537389; doi:10.1371/journal.pone.0313013)
Supplement: Attachment — Submitted filename: Plos One Reviewer Comments .docx [file pone.0313013.s005.docx]

Reviewer #1: This is an analysis of a carefully conducted pilot study to assess the feasibility of an exercise based intervention in a full-scale clinical trial. The feasibility study is well-designed, but the report is terse, and some of the details in the original published protocol referenced could be repeated. The manuscript is written in a conversational style that is sometimes off-putting: a patient "got COVID" (contracted), patients "did not mind", "to provide sufficient data to provide sufficient data", etc. A good editing with some more care to sentence structure and word choice is warranted.

Thank you for this point. The manuscript has been reviewed and the language has been edited.

One of the primary goals of a feasibility study is to provide information on the required sample size, which is given in the Discussion. This needs to be expanded to discuss the primary outcome, the hypothesis being tested, the test that will be used to test the hypothesis, and the assumptions that will be made gleaned from the feasibility study. Certainly attrition, retention, and adherence should be factored into this decision.

This is a great point. Thank you. I have included more depth in the discussion.

The randomization procedure used must be specified under CONSORT guidelines. 1:1 is not a randomization procedure. Permuted blocks? Complete randomization?

Thank you. I have added more information:

“Participants were randomized through simple random sampling using opaque envelops, by the lead student investigator, into either the Hands Up Program where they performed an online home whole body exercise and education program, twice a week for 6 weeks, or into the control group where they proceeded with their usual care. The allocation ratio was 1:1, with no stratification or permuted blocks”

You talk about correlated outcomes and how you might use the observed correlation to fine tune your decisions for outcomes. This needs to be talked about here. In fact, you don't discuss what types of outcomes would be appropriate for the full-scale trial; would you just have one primary outcome? What hypotheses have you decided would be of interest?

Thank you. I have included that in the discussion and results. I have also added more information about outcome measures in the methods:

“Additional outcome measures were assessed to gain insight into preliminary effects of the program. Secondary outcomes included gait speed, five times sit to stand, timed up and go, Biodex balance assessment, grip strength, pinch strength, wrist range of motion (pronation, supination, flexion, and extension), hip and waist circumference, bone mineral density, EQ5DL, patient-rated wrist evaluation (PRWE), disability of the arm, shoulder and hand (DASH), global rating of change scale (GRC), osteoporosis quality of life questionnaire (OQLQ), fear of falling questionnaire, and personalized exercise questionnaire. Participants in the intervention were also asked to participate in an exit interview to discuss their experience in the program.”

In results:

Consideration for Future Trial

A fully powered RCT with grip strength as the primary outcome(20) would require a sample size of approximately 350 participants. Our recruitment target was achieved in 14 months at this single site, with 272 participants that would have been eligible at the single site. Therefore, a larger trial at the single site might be accomplished in 5 years with the recruitment rate experienced in this study. To recruit more participants, quicker, to achieve the desired sample size in a larger trial, more sites could be included or we could increase the age criterion to all participants over the age of 50, or through potentially modifying the time commitment associated with engaging in the study.

Outcomes

Many secondary outcome measures were included in this study. Outcome measure burden was described as one of the key reasons participants withdrew from the study. Therefore it will be necessary to reduce the number of outcomes in a future larger trial. When looking at the Pearson’s Correlation Coefficients, the following outcomes has a strong correlation and therefore may be redundant: TUG and 5 times sit to stand (0.651), wrist flexion on the affected side and ulnar deviation on the affected side (0.626), wrist flexion on the affected side and radial deviation on the affected side (0.601), wrist flexion on the affected side and grip strength on the affected side (0.673), wrist extension on the affected side and ulnar deviation on the affected side (0.628), wrist extension on the affected side and radial deviation on the affected side (0.661), wrist extension on the affected side and grip strength on the affected side (0.641), ulnar deviation on the affected side and grip strength on the affected side (0.745), and radial deviation on the affected side and grip strength on the affected side (0.657).

The correlation table is uploaded as supplementary material.

In summary, a subsection of the results addressing statistical considerations of sample size and outcomes in the full-scale trial based on the feasibility trial would be a useful addition to this paper.

Thank you. I have added this subsection to the results.

Reviewer #2: The manuscript examines the feasibility of an exercise and educational intervention for patients 50-65 years of age, presenting with a DRF. The manuscript concluded that such a programme is feasible and should continue onto a larger trial.

I think there is merit to what has been done (the amount of work that has gone into the study is unquestionable) and I do not necessarily disagree with the conclusion. I do feel, however, that the manuscript lacks certain information that is essential in fully justifying the conclusions. I provide the key points below, with some explanation and I think consideration and addressing teh comments will improve the manuscript.

Introduction:
There is little information about the hands-up programme, whether in the manuscript or the study's registration. Some more information (it could even be in the Methods), would provide a sense of what the requirements are, what it does exactly, how it would be structured if not affected by Covid-19. This will provide context to the reader for the challenges described, the solutions etc. It would also help clarify apparent discrepancies - e.g. p5 Lines 92-93 and p5 Lines 99-100, are these the same things, are both being targeted by the intervention?
Thank you for this comment I have added more information to the introduction:

“There is little information conveniently accessible to people related to exercise, nutrition and fall prevention to reduce the risk of osteoporotic fractures.(10) Therefore we developed the Hands Up Program. The Hands Up Program targets individuals after they sustained a DRF and if they are between the age of 50-65. The intention of the program is compliment rehabilitation of the DRF with exercises and education that can address their risk of osteoporosis. The goal of this research is to develop and test a novel exercise and education group intervention (Hands-Up) that will incorporate education on bone health, prevention guidelines, nutrition and fall prevention, with an online exercise class and a structured virtual home safety assessment. The protocol has been previously published. The current study is addressing the feasibility of the program. Specifically, is the Hands-Up program a feasible intervention for patients aged 50–65 with a low-impact DRF?

This multimodal intervention will target the major risk factors for secondary fractures, to enhance functional recovery and health following a DRF, compared with usual physical therapy for wrist fractures.”

Methods:
There needs to be a statement re the study having received Ethical approval.

Sorry for that oversight. A statement on ethics has been added.
Heads-up - as above, if the Introduction is not the place to describe the intervention, then it is in Methods.

Thank you. I have added it to the introduction since the protocol has already been published.
Outcome measures - the outcome measures described in the manuscript, are 'half' of the ones described in the registration. Indeed, some physical measurements would help with the design of the larger trial, if nothing else to provide a sense of effect expected. Questionnaires were discussed, but not reported here. Interviews are reported, but not mentioned here. Perhaps linking with the 'more information for the intervention' comment, the rationale for the assessments should also be provided. For example, the measurements reported in the registration (e.g. handgrip strength, bone density) do not seem to align with the programme focusing on balance. Overall, the Methods make for an incomplete feasibility trial, while the Results appear to suggest that the necessary aspects were, in fact, present.

Thanks so much for this point.

I have added the following paragraph:

‘Additional outcome measures were assessed to gain insight into preliminary effects of the program. Secondary outcomes included gait speed, five times sit to stand, timed up and go, Biodex balance assessment, grip strength, pinch strength, wrist range of motion (pronation, supination, flexion, and extension), hip and waist circumference, bone mineral density, EQ5DL, patient-rated wrist evaluation (PRWE), disability of the arm, shoulder and hand (DASH), global rating of change scale (GRC), osteoporosis quality of life questionnaire (OQLQ), fear of falling questionnaire, and personalized exercise questionnaire. Participants in the intervention were also asked to participate in a semi-structed exit interview to discuss their experience in the program. The interviews were recorded and transcribed verbatim. The interviews were analyzed using interpretive descriptive methods(13) to identify key themes to improve the Hands-Up Program. Participants were prompted to discuss their general impression of the program, the exercise portion, the education portion, the outcome measures, whether they would continue the program and any additional thoughts.’

Results:
There were no results presented for the usual care group e.g. adherence, follow-up etc. It is hard to assess whether the Hands-up programme was eg un/feasible purely on the thresholds set, without comparing the findings to what happens with usual care. If, for example, usual care follow-up is 40% and hands-up is 60% that appears better than usual care, even if the threshold of e.g. 70% is not achieved.

This is a really great point and something that we could do a better job of tracking in the larger trial. I’ll mention it in the discussion and limitations.

Discussion:
Partially affected by the lack of information on the intervention, the recommendations made are not supported. Conjectures are presented frequently, as early as the Results ('..so other injuries may have occurred and were not reported..'). Several recommendations appear to have no basis for the suggested change; would that not end up becoming another feasibility trial? The evidence from the interviews would be useful in a more robust presentation regarding the outcomes, which would inform the subsequent trial.
Several discussion points appear to be contradicting, for example a) Covid-10 forced to online delivery which was not ideal as in-person delivery would be better vs an issue was the time commitment and travelling to the hospital (which would increase with full in-person delivery), b) increase the age bracket for better recruitment vs the actual aim of the study as described in the Introduction.
More clarity is needed throughout the section, for example a) novel exercise and education group intervention including education, prevention and nutrition vs enhancing functional recovery and health vs strength as an assessment criterion; how do these align.
Was the time commitment to the travel, the completion of the questionnaires or both the barrier (P13, Lines 274-276)?

Thank you for these thoughtful comments. I have done my best to address them throughout the discussion.

Overall, I think there is merit in the study. In my view, however, it requires considerable revision to address the above issues, before it can be recommended for publication.
