# Supplementary material for: Hands Up Program: Results of a feasibility study of a randomized controlled trial of a bone health exercise and education program for adults aged 50–65 post distal radius fracture
Source: PLoS One. 2024 Nov 5;19(11):e0313013. doi: 10.1371/journal.pone.0313013 (PMC11537389; doi:10.1371/journal.pone.0313013)
Supplement: Attachment — Submitted filename: plos one reviewer comments 2 .docx [file pone.0313013.s006.docx]

6. Review Comments to the Author

Reviewer #2: The authors have attempted to address my previous comments; this was successful at some points, while less so in others. I will try and discuss further the remaining points, with the aim to provide more clarity on the issues I think require attention. The more key ones are presented first.

Discrepancies throughout: there are still discrepancies in the manuscript. The selection of the age range is based on the thought processes, evidenced in the Introduction. To then recommend increasing the age range or stating that the 'age restriction.. reduced the.. participants', questions the rationale for those decisions. Further, increasing the age range potentially induces different issues (assuming an age-based digital divide, if the age range is increased to younger people, the difficulty in online assessment may be less, while if it increased to older people, the difficulty in online assessment may be more; transportation maybe less / more of a problem; time availability might be more / less of a problem (e.g. if >65 are retired) etc). Therefore, the solution can not be to alter parameters that would affect the process described.

This is a great point. I have removed the suggestion of increasing age range.

Aim of the study: the aim of the programme is described as 'to complement rehabilitation of the DRF'. Its possible implementation is not clear. The description appears to present is as a standard addition to the usual care, while it appears it could be an option. In that respect, will it work for those who choose that option? Why is there insistence on a single site when travelling was reported as an issue, and delivering online at a different site might increase the uptake (close enough for the in-person visits, and online so they are reduced). There seems to be a need for a clearer 'so what' in the study.

I don’t believe there is insistence on a single site. I reported that we only collected from one site for this pilot study but suggested adding additional sites would help support reaching our sample size target for a fully powered RCT. I fully agree that adding more sites can increase the uptake as well. I’ll make sure that’s more clear in the discussion.

Recommendations: There are several recommendations that seem unsupported and would alter the parameters of the feasibility trial. Please revisit and address accordingly. Perhaps a helpful starting point that would help with all of the above is Lancaster and Thabane (<https://pilotfeasibilitystudies.biomedcentral.com/articles/10.1186/s40814-019-0499-1>). This would help with providing a clearer direction.
Thank you for this recommendation. I reviewed the CONSORT reporting guidelines and reviewed the extension for non-randomized trials, despite this being a randomized trial.

Specific comments
Page 5, Line 95: please replace 'is compliment rehabilitation' with 'is to complement rehabilitation'

Thank you, done.

Page 5, Line 102: Please convert the question to statement.

Done.

Page 6, Line 116 - the ratio has already been reported earlier

This has been removed.

Page 6, Line 120 and Page 6, Line 128: Please keep one of the two statements re Ethics. Also add the Body that provided the Ethical approval

Thank you. Ethics approval is only stated once, but that it was approved by the Western Research Ethics Board.

Page 6, Line 122 - maybe useful to indicate to the reader that the Outcome measures are appearing later on

Thank you, done.

Page 7, Line 149-150 - were the intervention group not starting the intervention at the same time point, i.e. after cast removal? Please make very clear the time points for the intervention group, as this may impact on whether there were other parameters introduced (linking also to a statement made on page 11, Line 221).

Good point. I have added clarification that the intervention group started after their cast was removed.

Page 8, Line 164 - ideally, the ones that did not participate would also offer ideas as to why they have not.

That’s a good point. Unfortunately we did not interview participants in the control group.

Page 8, Line 174 and Page 9, Line 175 - please remove the duplication

Thank you. The a priori success criteria was removed, as it was all captured in the outcome measure section.

Page 8, Line 175 - where is this analysis stemming from? No such 'plan' was described, nor justified by the Introduction. No discussion was present around sex- / gender-based differences. Further, no such analysis was conducted (or at the very least, presented).
Statistical analysis: The reason for the correlation testing should be included here. It exists in the Results but it should be here; to enable reduction of the outcome measures. There is also no description of how the qualitative data will be analysed.
Results: The qualitative results should be better analysed and described. Perhaps a helpful starting point that would help with all of the above is Anderson (<https://www.ncbi.nlm.nih.gov/pmc/articles/PMC2987281/>) and Braun and Clarke (<https://www.tandfonline.com/doi/abs/10.1191/1478088706qp063oa>).

Thank you for this feedback. I have addressed this in multiple places throughout the manuscript.

Page 13, Line 265: Why were they not captured, the participants recorded health-related events? It also contradicts Page 15, Line 313.
Considerations for future trial: this should appear in the end, when all parameters have been presented to the reader.

This is a good point. I have removed that adverse events weren’t captured, as they were.

Outcomes: The criterion for removing redundant outcome measures was an R>0.8 ; none of the proposed ones me that criterion.

Good point. I have clarified that

Outcome measures: In the table, you should present the results at all time points, not only at baseline.

Thank you. The additional time points have been added.

Page 14, Lines 305: how was this sample size calculated?

More of a description has been added.

Page 15, Lines 313-316 - not sure of the purpose of this para; no adverse effect were observed (albeit earlier it was stated they were not formally captured), so why is the reference important?

This paragraph was deleted.

Page 18, Line 379 - please replace 'it's not' with 'it is not'

Done.

Discussion: as per the main comment earlier, it needs clarification and better structure to convey the results, considering the actual aim of teh study ('so what') and the purpose of a feasibility study.

Good point. I have tried to streamline the discussion to make it link more to the feasibility study and discussion the potential for the future trial.
